# Unilateral Hyperpigmented Facial Lesion Since Birth

**DOI:** 10.7759/cureus.47901

**Published:** 2023-10-29

**Authors:** Kalyan Dalave, Shreya Deoghare, Yash Buccha

**Affiliations:** 1 Department of Dermatology, Venereology and Leprosy, Dr. D. Y. Patil Medical College, Hospital and Research Centre, Dr. D. Y. Patil Vidyapeeth, Pune, IND

**Keywords:** becker's nevus syndrome, becker's melanosis, irregular network of dark brown pigment, epidermal hyperpigmentation, becker's nevus

## Abstract

Facial hyperpigmented lesions that are unilateral are a rare and challenging dermatological anomaly since birth which can be genetic and non-genetic. This paper seeks to provide an exhaustive overview of the etiology, clinical presentation, differential diagnosis, and management strategies for these congenital lesions. Unilateral facial hyperpigmented lesions can be caused by a number of conditions, such as congenital melanocytic nevi, Becker's nevus, nevus of Ota, linear epidermal nevi, and café-au-lait macules. Accurate diagnosis requires meticulous examination, dermoscopy, and histopathological evaluation. Observation, topical therapies, surgical excision, and laser therapy are among the available treatment options. Treatment decisions should be influenced by factors such as lesion characteristics, aesthetic concerns, and patient preferences. Long-term supervision and psychosocial support are indispensable elements of comprehensive management. We present a case of a 12-year-old patient with a progressively growing hyperpigmented lesion on the right side of the face, present since birth with an intermittent area of normal skin in between. Dermoscopy unveiled an irregular, dark brown pigment network, and histopathological evaluation showed an increased number of melanocytes in the dermis. This case highlights the diagnostic challenges of such lesions and underscores the significance of a multidisciplinary approach for accurate evaluation and management. This paper aims to cover existing knowledge gaps regarding unilateral facial hyperpigmented lesions since birth and direct future research efforts for diagnostic and therapeutic interventions.

## Introduction

A dermatological disorder known as hyperpigmentation of the skin is characterized by a change in the color of the skin, which can be described as either a darkening or a discoloration compared to normal skin. The therapies for hyperpigmentation problems typically take an extremely lengthy time to show benefits and have a low compliance rate among patients [1]. A birth lesion raises important considerations about its etiology, differential diagnosis, and treatment. Congenital dermatological anomalies like hyperpigmented lesions on one side of the face are popular and have clinical implications. These lesions vary in size, shape, and color, resulting in several clinical symptoms. To correctly identify and treat these lesions, one must first grasp their origins and potential consequences. Unilateral facial hyperpigmented lesions are difficult to diagnose due to the large range of diseases that might show similarly [2]. Differential diagnoses include congenital melanocytic nevi, Becker's nevus, Ota's nevus, linear epidermal nevi, and others. To distinguish these illnesses, a thorough examination is needed. These lesions also have a significant emotional impact on those affected. Hyperpigmented facial lesions can shame, isolate, and lower one's self-esteem. Thus, early discovery, precise diagnosis, and appropriate management are essential for medical and psychological reasons. Unilateral facial hyperpigmented lesions from birth can be treated under certain conditions depending on the lesion size, location, and patient preferences. Treatment options include observation, topical medicines, surgical excision, and laser treatments [3]. The treatment options' risks, advantages, and cosmetic consequences must all be taken into consideration while selecting the treatment option. This study illuminates the clinical appearance, differential diagnosis, and treatment options for unilateral facial hyperpigmented lesions present since birth. The case study will examine and evaluate the relevant literature to better understand these dermatological conditions and help develop evidence-based guidelines for their treatment. Addressing these lesions should improve patient results, psychosocial suffering, and cosmetic outcomes. This case study can pave the way for future studies on the genetic, environmental, and molecular factors that cause highly pigmented lesions on the face from birth.

## Case presentation

**Figure 1:**
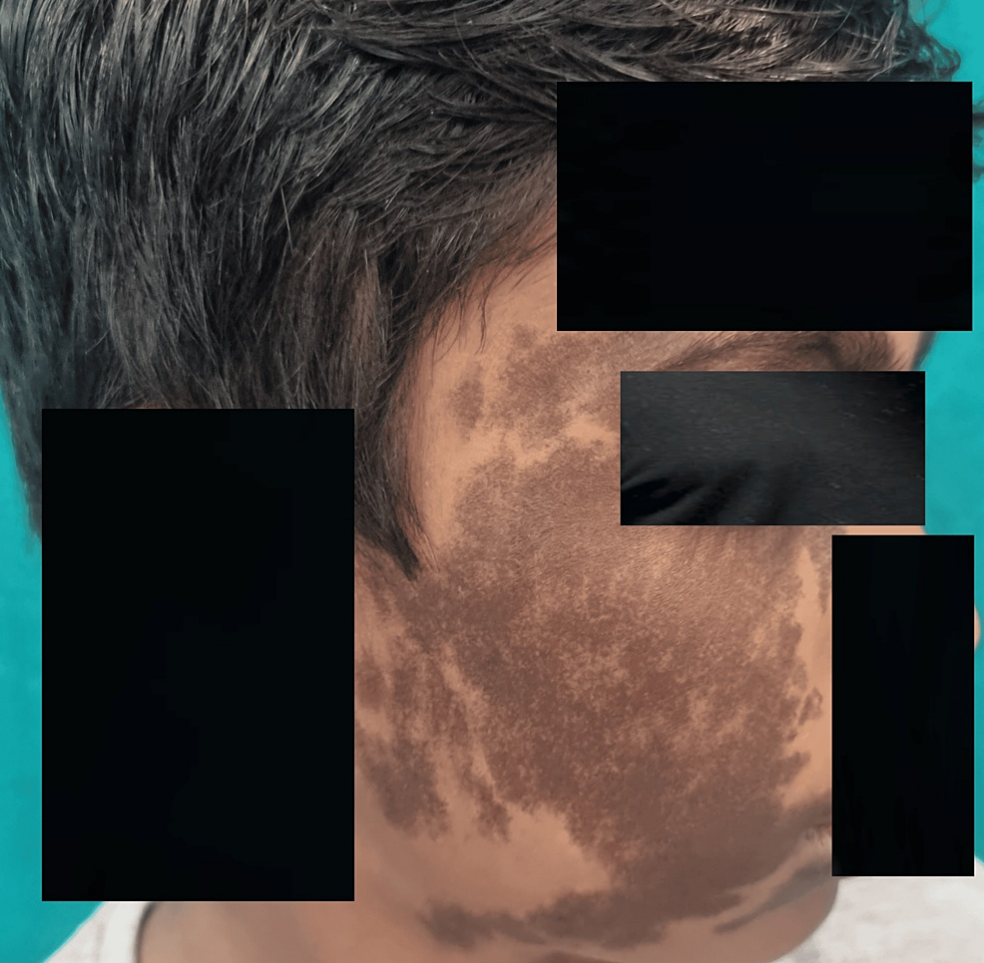
Hyperpigmented brownish patches present on the right side of the face since birth

**Figure 2:**
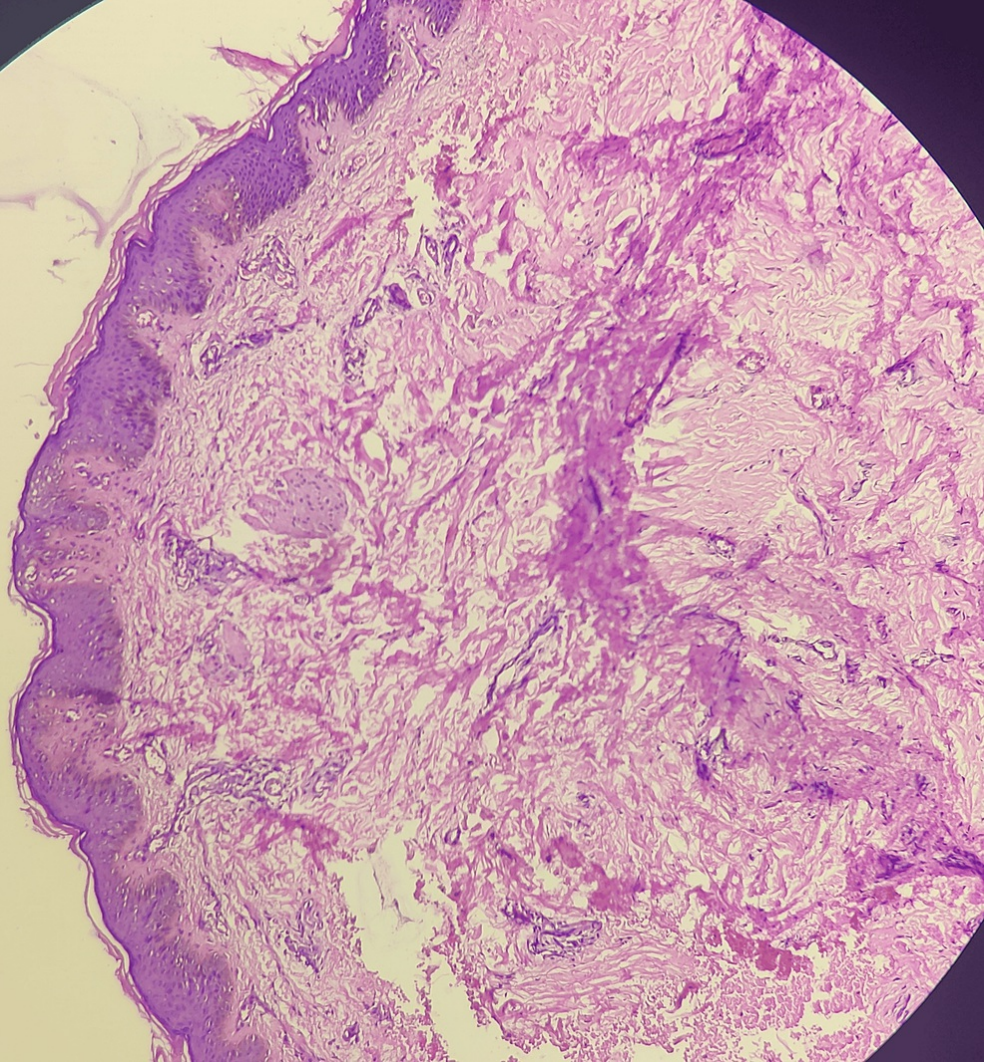
Hyperkeratosis and hyperpigmentation of epidermis (H&E, 40x)

A 12-year-old boy was diagnosed with a dark-colored, flat lesion that had been present from birth on the right side of his face which was progressively growing in size. The patient disputes having ever had a burn, been exposed to chemicals, or used any cream in the past. Upon inspection, the right forehead, periorbital, cheeks, and perioral areas displayed unilaterally present hyperpigmented brownish patches with sporadic areas of normal skin in between (Figure 1). Dermoscopic examination (DermLite DL4, polarized light) revealed an irregular, dark brown pigment network. Histopathological analysis with hematoxylin and eosinophilic stain demonstrated hyperkeratosis and hyperpigmentation of the epidermis, along with an increased number of melanocytes in the dermis (Figure 2).

To prevent the growth of hair on the pigmented area and to reduce the pigmentation on the face, the patient is undergoing the treatment of alternate sittings of 808nm diode for reduction of hair and Q-switched Nd:YAG laser at 1064 nm for pigmentation respectively in a gap of 15 days. After three sittings of each laser, no visible hair is present and the pigmentation has reduced as experienced by the patient. The patient is still on follow-up.

## Discussion

Hyperpigmentation darkens the skin more than the rest of the skin. Melanin, which gives skin its color, is produced in excess. It can affect any skin type and is more likely to arise during pregnancy, aging, or injury. The unilateral facial hyperpigmented lesion is a serious issue for diagnosing and treating in the field of dermatology. These lesions, which darken one side of the face, can lower the quality of life [4]. Unilateral facial hyperpigmented brownish spots are an uncommon dermatological condition darkening one side of the face. These patches which vary in size, shape, and color can affect one's looks and mental health. Histological diagnosis problems and malignancy bias debate melanoma incidence and congenital melanocytic nevus (CMN) treatment. A CMN is a benign nevomelanocyte lesion. Huge, hairy nevi have abundant hair development. Larger lesions are riskier for melanoma and cosmetic issues, harder to surgically remove, and have more symptoms. CMN's genetics may diagnose, estimate risk, and treat melanoma. Oral mitogen-activated protein kinase inhibitors treat neuroblastoma RAS-mutated CMN patients with probable cutaneous and central nervous system melanoma [5]. The anterior chest, shoulder, scapular area, and upper arm had right-side hyperpigmentation, hypertrichosis, and irregular borders. The lesion grew into large regions. Histology revealed upper dermis melanophages and basal layer hyperpigmentation. Clinical and histological studies indicated Becker's nevus as the cause.

Dermatologists have struggled to diagnose unilateral facial hyperpigmentation with brownish patches since birth [6]. Treatment options depend on a variety of circumstances. Because of the psychological and physiological ramifications of these issues, they must be identified early, correctly diagnosed, and addressed quickly. This condition requires more investigation to determine the cause, provide better treatment choices, and enhance outcomes [7].

An uneven network of dark brown pigment is a common dermatological characteristic that can be found in a range of skin conditions. It is a word that refers to an aberrant pattern of pigmentation that may be seen when the skin is examined visually [8]. A dark brown pigment network can be seen in a number of skin conditions, including melanocytic lesions like melanoma and atypical nevi and non-melanocytic conditions like seborrheic keratosis and pigmented basal cell carcinoma [9].

Pigmented basal cell carcinomas are significantly less prevalent than their non-pigmented counterparts. During the dermatological examination, non-pigmented basal cell carcinomas can be easily differentiated from other types of skin lesions due to their asymmetrical arborizing vessels, pink color, and focal ulceration [10].

Clinicians may face difficulties in making accurate diagnoses if they are unable to determine the relevance of mucosal-pigmented lesions. When the cause of the pigmentation cannot be diagnosed with absolute certainty based on the clinical presentation, a biopsy is recommended as the next course of treatment [11]. Clinical presentation and histological findings help make the diagnosis. Even though the illness usually cures on its own, uncontrolled pruritus and extensive mucosal erosions cause significant morbidity.

Becker nevus syndrome is a phenotype characterized by the presence of Becker's nevus with ipsilateral breast hypoplasia or other cutaneous, skeletal, and/or muscular disorders. This is one of the syndromes that comprise epidermal nevus syndrome, which typically begins at birth and intensifies substantially during adolescence. Compatible with Becker's nevus, the macule's histopathology revealed moderate acanthosis, hyperpigmentation of the basal layer with irregular granules of melanin, the presence of melanophages in the papillary dermis, and hyperplasia of the arrector pili muscle. Becker's nevus is a hyperpigmented hamartoma with hypertrichosis and well-defined borders that can appear anywhere on the body but is most prevalent on the trunk and upper extremities. Person and Longcope discovered an increase in androgen receptor levels in Becker's nevus in 1984. Later studies corroborated this increase, leading to the hypothesis that this cutaneous hamartoma is hypersensitive to hormones. Some patients with acneiform lesions of Becker's nevus have been reported in the literature, and it is hypothesized that this lesion may be mediated by androgens [12]. The cutaneous appearance of epidermal nevi is influenced by the predominant cell type, the age of the patient, the affected body region, and the degree of cell differentiation. Epidermal nevi adhere to Blaschko's guidelines. Schimmelpenning and later Feuerstein and Mims first described the association between epidermal nevi and abnormalities of the central nervous system [13]. If there are a lot of extracutaneous lesions and the epidermal nevus is spread out, it is more likely that the central nervous system and other parts of the body will be affected. This may be a sign of how bad the biological inheritance caused by the epidermal nevus is. In the vast majority of cases, the syndrome occurs randomly, and a familial link has rarely been identified. Individuals with a heterozygote genotype would be phenotypically normal, and the responsible allele would be passed down through several generations.

## Conclusions

Dermatologists have struggled to diagnose unilateral facial hyperpigmentation with brownish patches since birth. Depending on several factors, treatment options include observation, topical therapies, surgical excision, and laser therapy. These problems' psychological and physiological implications require early recognition, correct diagnosis, and rapid action. Unilateral facial hyperpigmented brownish spots that have existed since birth need more research to establish the source, provide better treatment options, and improve results.
